# Morphogenetic Factors as a Tool for Enhancing Plant Regeneration Capacity During In Vitro Transformation

**DOI:** 10.3390/ijms26178583

**Published:** 2025-09-03

**Authors:** Semyon D. Bakulin, Sokrat G. Monakhos, Sergey A. Bruskin

**Affiliations:** 1 Department of Molecular Breeding, Cell and Seed Technology, Russian State Agrarian University—Moscow Timiryazev Agricultural Academy, 127550 Moscow, Russia; bakulinsd@yandex.ru (S.D.B.); s.monakhos@rgau-msha.ru (S.G.M.); 2 Vavilov Institute of General Genetics, Russian Academy of Sciences, 119991 Moscow, Russia

**Keywords:** morphogenetic transcription factors, embryogenesis, meristem formation, organogenesis, *WUSCHEL*, *BABY BOOM*, *GRF-GIF*, transformation, tissue culture

## Abstract

Morphogenetic factors (MTFs) are specialized plant genes and transcription factors that play pivotal roles in embryogenesis and organogenesis. This review focuses on their functions in plant development regulation and their applications in plant biotechnology and modern breeding. Common challenges in transformation and regeneration were discussed, along with successful case studies demonstrating improved regeneration capacity and transgene stability in rice (*Oryza sativa*), soybean (*Glycine max*), rapeseed (*Brassica napus*), tomato (*Solanum lycopersicum*) and other less common crops and plant model organisms. These improvements were achieved through the utilization of key developmental MTFs such as *WUCHEL*, *BABY BOOM*, *GRF-GIF*, etc. The principles of designing genetic constructs with MTFs are explored, including promoter selection and regulatory elements, as well as their synergistic effects with phytohormones like auxins and cytokinins for optimizing in vitro morphogenesis. Current limitations in MTF expression and strategies to overcome them are analyzed. The article highlights recent advances, including MTFs potential for developing stress-resistant, high-yielding cultivars. Key discussion points include the discovery of novel morphogens, their application to recalcitrant species, and prospects for expanding the range of easily transformable and regenerable crops. Future directions involve developing universal transformation protocols and integrating morphogens with precision genome editing technologies, offering new opportunities for agriculture and global food security.

## 1. Introduction

Plants genetic transformation represents a broad group of methodologies widely used today in both fundamental research and practical breeding applications. The integration of tools such as CRISPR/Cas with plant transformation methodologies allows for the accurate and targeted modification of plant genomes, including those of economically valuable crop varieties. This approach allows targeted modification of specific traits or trait groups while minimizing potential negative effects on other valuable agricultural characteristics [1].

Successful transformation examples date back to the 1980s, with the first transgenic plants of soybean (*Glycine max* L.) [2], cotton (*Gossypium hirsutum* L.) [3], rice (*Oryza sativa* L.) [4], tomato (*Solanum lycopersicum* L.) [5], and rapeseed (*Brassica napus* L.) [6]. Transgenic varieties of other key crops followed in the 1990s: maize (*Zea mays* L.) [7], wheat (*Triticum aestivum* L.) [8], sorghum (*Sorghum bicolor* (L.) Moench) [9], and sugarcane (*Saccharum officinarum* L.) [10].

Parallel developments occurred in plant tissue culture techniques in vitro culture techniques, particularly in optimizing regeneration of transformed tissues. Fundamental work by F. Skoog and C. Miller [11] established the crucial role of phytohormones in regulating plant tissue growth and development. Subsequent studies by various researchers explored transformation protocols and phytohormonal regulation in different plant species [12,13,14]. Despite progress, challenges persist in achieving efficient transformation and regeneration for most agricultural cultivars [15,16,17,18]. The complex and multifactorial process of transformation shows a strong genotype dependence, with many protocols failing to work when applied to new varieties, despite working reliably with long-established model cultivars [19,20,21].

Beyond classical phytohormonal approaches, emerging strategies employ transcription factors that activate morphogenesis pathways (morphogenesis regulators/morphogens) [13].

## 2. Morphogenetic Factors: Definition and Classification

MTFs typically refer to plant genes whose ectopic expression can initiate morphogenesis. These genes most often encode transcription factors—the “master switches” of development. To date, a broad spectrum of such genes with diverse functions and origins has been discovered [22]. The principal classes of MTFs include the following groups.

### 2.1. WOX Factors (WUSCHEL-Related Homeobox)

A family of homeodomain transcription factors responsible for maintaining meristem activity. The key representative is the *WUSCHEL (WUS)* gene, first identified in *Arabidopsis thaliana* (L.) Heynh. [23]. It is essential for shoot apical meristem function: *WUS* stimulates meristem cell proliferation while preventing their differentiation, thereby sustaining meristem activity. Mutations in *WUS* lead to meristem depletion and shoot growth arrest [24]. Studies show that *WUS* overexpression can induce embryoid formation directly on vegetative organs in adult plants [25]. For example, introducing *WUS* into *Arabidopsis* leaves triggered somatic embryogenesis, enabling plant regeneration without phytohormonal regulators [26,27]. Today, *WUS* from *Arabidopsis* and rapeseed is increasingly used to enhance regeneration capacity across recalcitrant species like coffee (*Coffea canephora* Pierre ex A.Froehner) [26], orchids (*Phalaenopsis* Blume) [27], banana (*Musa acuminata* Colla) [28], cotton [29], maize [30] sorghum [31].

*WOX* genes regulate meristem cell differentiation, root meristem growth, and somatic embryogenesis, improving regeneration efficiency [32,33]. For instance: *WOX13* is critical for callus cell differentiation in *A. thaliana* [34]; *WOX4* participates in wood formation, while *WOX11* and *WOX12* mediate salt and water stress responses [35]; *WOX11/12* determine root meristem cell fate in *A. thaliana* [36]; *WOX5* promotes root initiation in other plants [37,38].

### 2.2. BBM (BABY BOOM)

This *APETALA2*-like (*AP2*-like) transcription factor was discovered during studies of somatic embryogenesis in rapeseed [39]. The constitutive expression of rapeseed *BnBBM* in *Arabidopsis* induced massive somatic embryoid formation on vegetative tissues, such as leaves and shoot apices, etc. These embryoids developed into intact plants, even without the use of phytohormones [39]. This demonstrated that a single gene could activate the full embryonic program in somatic cells. Subsequent studies has confirmed the universality of *BBM*: similar effects were observed in tobacco (*Nicotiana tabacum* L.) [40], soybean [41], cacao (*Theobroma cacao* L.) [42]. Functionally, *BBM* and related *AIL (AINTEGUMENTA-like*) factors maintain embryo and young organ meristem activity by stimulating cell division and initiating embryogenesis.

### 2.3. PLT Factors (PLETHORA)

*PLT5* in *Arabidopsis* contributes to root meristem formation. Overexpression enhances callus and shoot regeneration from stem wounds [43]. Like *BBM*, *PLT* actors induce embryogenesis but primarily influence root pole development, making them promising regeneration enhancers.

### 2.4. GRF-GIF Gene Family

This paired module consists of a growth-stimulating transcription factor (*GRF*) and a stabilizing cofactor—*GRF*-interacting factor (*GIF*). The co-expression of *GRF-GIF* fusion proteins significantly enhances regeneration. For example, *GRF4-GIF1* significantly increased wheat regeneration efficiency by 8-fold, allowing for genotype-independent transformation [44]. In soybean, the expression of *GmGRF-GIF* allowed the transformation of previously resistant cultivars [45]. Heterologous *AtGRF5* expression enhanced the recovery of transgenic melons (*Cucumis melo* L.) [46]. Unlike *WUS/BBM*, which induces direct embryogenesis, this strategy promotes the general growth of meristems to facilitate regeneration.

### 2.5. Other Morphogenetic Factors

*LEC1/LEC2 (LEAFY COTYLEDON)* are embryo maturation factors that rejuvenate cells and promote somatic embryogenesis. The effect of this MTF has been observed in tobacco, during the process of dedifferentiation from immature pollen into embryogenic cells, as well as in *Arabidopsis*, during somatic embryogenesis [47,48] and embryo development [49]. *SERK1* (*Somatic Embryogenesis Receptor Kinase 1*) is a receptor gene marking embryogenic competence; its overexpression leads to an increase in the yield of somatic embryos [25]. *ESR1* (*Enhancer of Shoot Regeneration 1*) is a factor that enhances *Arabidopsis* shoot formation in vitro [50,51] sometimes in synergy with *WUS* [52]. *WIND1* (*WOUND INDUCED DEDIFFERENTIATION1*) is a wound-induced factor initiating dedifferentiation via cascaded morphogen expression in *Arabidopsis* [53,54,55] and tobacco [55]. *RKD (RWP-RK domain*) is a gene family that is involved in nitrogen response [56] and gametophyte development [57], as well as the development of symbiotic root nodules in the Fabaceae family [58] and embryogenesis in monocot plants [59]. *DOF* (*DNA-binding One Zinc Finger*) are root/shoot meristem regulators (e.g., *TaDOF3.4, TaDOF5.6* in wheat) [60].

Some *Agrobacterium* (syn. *Rhizobium*) oncogenes exhibit morphogenesis effects: *ipt* (*isopentenyltransferase*) (from Ti-plasmids) encodes cytokinin synthase, inducing shoot branching [55,61,62]; *rolB/rolC* (*root loci* from *A. rhizogenes*) alter hormonal balance to stimulate root or shoot meristem activity [13].

REF1 (REGENERATION FACTOR1) Peptides. These wound-responsive peptides bind *PORK1* (*PEPR1/2 Ortholog Receptor-Like Kinase1*) receptors to activate *WIND1* and regeneration, as demonstrated in tomato, wheat, maize, and soybean [63].

MTFs often cause developmental abnormalities if constitutively expressed [13]. Thus, their activity must be tightly controlled [24]. Key MTFs used in plant biotechnology are summarized in Table 1.

## 3. Case Studies of Morphogenetic Factors’ Utilization in Crop Transformation and In Vitro Regeneration

The use of MTFs is becoming more and more common in transformation and regeneration protocols for various plant species. However, most of these experiments remain basic or even theoretical [15]. These factors are especially valuable for difficult-to-transform crops with low efficiency at the species or variety level, as well as for important commercial plants. Extensive studies on the use of morphogens have been conducted with rice, rapeseed, soybeans, and tomatoes. The detailed applications of various MTFs are presented in Table 2.

### 3.1. Rice

Among Poaceae, rice was the first crop successfully transformed [100,101]. Most high-efficiency transformation reports (up to 90%) involve *O. sativa subsp. Japonica* cultivars [20,102], typically using scutellum-derived callus [103]. By contrast, *Indica* subspecies (e.g., *O. sativa* subsp. *indica*) exhibit poor regeneration due to low morphogenesis-related gene activity [20], hypersensitivity to phytohormone ratios, and phenolic compound accumulation [104].

Co-overexpression of maize *WUS* and rice *BBM* enhances somatic embryogenesis and meristematic activity, dramatically improving regeneration rates. *Indica* rice, *OsBBM* + *ZmWUS2* yielded transgenic shoots in 43% of cases (vs. ~3% in controls), while *ZmBBM* + *ZmWUS2* achieved 27% efficiency [15]. The regulatory module *uORF-HsfA1a-WOX11* fine-tunes *WOX11* expression, enabling root system modifications without affecting shoot traits [72].

The *GRF4-GIF*1 module significantly doubles the regeneration efficiency in the ‘Kitaake’ cultivar, producing fertile plants without any developmental abnormalities, unlike constitutive *WUS/BBM* expression [44]. *AtGRF5* orthologs also boost transformation rates in monocots, including rice [20].

For recalcitrant black rice (*O. sativa* ‘Cempo Ireng’), ectopic expression of *OsRKD3* induced somatic embryogenesis by activating a gene network (*AP2/ERF*, *MYB*, *COL*) and hormonal regulators, restoring near-zygotic totipotency [59].

### 3.2. Rapeseed

Rapeseed was first transformed by Moloney [6]. Today rapeseed cultivars like ‘Westar’ [65,105] and ‘ZS11’ [106,107] achieve 50–90% transformation efficiency. However, winter cultivars requiring vernalization (like ‘Express617’) remain highly recalcitrant [65].

*WUS* and *BBM* are widely used to improve plant regeneration. While standard methods result in less than 1% transgenic shoot formation in ‘Westar’, overexpression of *BnBBM* stimulates the development of somatic embryos in the absence of exogenous phytohormones [39]. Sugar beet *WUS* enabled transgenic shoot regeneration in winter rapeseed, bypassing callus formation. However, prolonged *WUS* expression may cause abnormalities [15]. *AtGRF5* increased shoot regeneration to 19.6% (vs. 3% in controls) without developmental defects [20].

No publications are known regarding the use of other MTFs for rapeseed transformation. For other Brassicaceae species like *Arabidopsis* combining factors (e.g., *WUS* + *STM*) is promising: their co-expression generated meristems and floral structures in vitro [67]. *WIND1*, *RKD*, *PLT*, and *ESR1* are also candidates for rapeseed [51,65,77].

### 3.3. Soybean

A notoriously difficult-to-transform crop, soybean was first modified via biolistics [108], with *Agrobacterium*-based methods lagging due to complexity [109]. Challenges include low efficiency, genotype dependence, and laborious protocols via biolistics [108] and cotyledonary nodes from mature seeds [110]. Reliable methods exist only for lab cultivars (e.g., ‘Williams 82′), while elite varieties remain recalcitrant: ‘Heinong44′ [111], ‘Shennong 9′, ‘Bert’ [112], ‘BR-16′, ‘BR-19′ and others [113].

*GmGRF3-GIF1* increased regeneration up to 5.5–13.8% (vs. 2–5% in controls) in previously untransformable cultivars, reaching 16.7% with *GRF3* mutations [45]. *AtGRF5* achieved 50% shoot regeneration in ‘Jack’ and ‘CD215′ [20]. *BBM* + *WUS* triggered somatic embryogenesis, and *BnBBM* enhanced transformation [43]. *WIND1*, *LEC2*, and *ipt* are also promising, as demonstrated in tobacco [53,68,77], tomato [77], poplar [114].

### 3.4. Tomato

Easier to transform than soybean or rice, tomato protocols date to 1986 [115,116]. Yet genotype-specific issues persist: low *WUS/CLV* (*CLAVATA*) expression [117], phytohormone sensitivity [118], *Agrobacterium* resistance [119], somaclonal variation [120], and selective agent toxicity [121].

*PLT5* and *WUS* enabled in planta transformation with 13% regeneration (vs. 0% in controls). It has also been observed in snapdragons (*Antirrhinum majus* L.) through *Agrobacterium* injection. Enhanced transformation and shoot regeneration of turnip (*Brassica rapa* L.), as well as the formation of transgenic calli and somatic embryos in sweet pepper (*Capsicum annuum* L.) have been observed [43]. The peptide REF1 also induced callus-to-shoot morphogenesis [63].

Several examples are known of using other MTFs for transformation in species of the Solanaceae family. Tobacco (*Nicotiana*) experiences enhanced regenerative capabilities through the action of *WIND1* [53], *LEC2*, and *ipt* [77], while pepper (*Capsicum*) species rely on *BBM* for regeneration [25,73]. The use of these morphogens in tomato may also enhance its in vitro regenerative capacity.

### 3.5. Other Crops

There is known data on the use of MTFs to induce regeneration processes after transformation for other common or more exotic crops or model plant species.

Some of the most challenging crops are fruit trees, such as *Citrus* L. species. For certain *Citrus* species, MTFs have been used to overcome regeneration bottlenecks. For instance, the gene *L1L* (a homolog of *LEC1*), when overexpressed in sweet orange (*Citrus × sinensis* (L.) Osbeck) and tangerine (*Citrus reticulata* Blanco), induced embryoid formation as early as one month after *Agrobacterium*-mediated transformation [83]. Interestingly, in *Citrus*, some MTFs can also enhance transformation efficiency itself. For example, in lemon (*Citrus limon* (L.) Osbeck) and sweet orange, overexpression of *ZmKN1* under the 35S promoter increased *Agrobacterium*-mediated transformation efficiency by 3–15 times [84]. In the study by Debernardi et al. [44], the use of *GRF-GIF* was demonstrated to enhance regeneration in citron (*Citrus medica* L.) by 4.7-fold. It is known that *Citrus* species are considered quite challenging subjects, both for transformation and general in vitro cultivation, such as in micropropagation [122,123,124].

Other fruit tree crops are also challenging for gene editing. However, even for these species, there are some—albeit limited—successful examples of improving regeneration capacity through morphogens.

For example, in the case of the apple plant (*Malus domestica* L.), which is known to be a difficult system for in vitro transformation, preliminary transformation of leaf explants with the *MdBBM1* gene resulted in an increased frequency of transgenic shoot regeneration of approximately 31%, which is significantly higher than the standard range of 5–10% for this species. The study by Chen et al. [85] demonstrated that *BBM* expression in apples accelerates regeneration initiation and increases the number of regenerant plants without compromising their development. The *BBM* cassette was subsequently removed, yielding transgenic plantlets free of morphogenic genes. This was the first successful application of a *BBM*-type gene in a fruit tree.

Grapevine (*Vitis vinifera* L.) is another highly relevant crop for gene editing. In one study, the regeneration efficiency of the ‘Cabernet Sauvignon’ cultivar from embryogenic culture was increased to 42.85% using *VvBBM* [86]. Additionally, the use of *GRF-GIF* in grapevine improved shoot regeneration efficiency by more than 4-fold [44].

Among other berry crops, the application of morphogens has so far only been reported in strawberry (*Fragaria vesca* L.). For example, the grape-derived *VvGRF4-GRF1* enhanced strawberry transformation efficiency by more than 2-fold, reaching 40% [87]. For commercially important berry crops such as raspberry (*Rubus* subg. *Idaeobatus* (Focke) Focke), blackberry (*Rubus* subg. *Rubus* L.), bilberry (*Vaccinium myrtillus* L.), and blueberry (*Vaccinium corymbosum* L.), there are no known studies yet on the use of MTFs. However, this approach could hold significant promise for genomic selection in these species.

Among woody plants, the successful application of MTFs in cacao (*Theobroma cacao* L.) is particularly noteworthy. In this species, the endogenous gene *TcBBM* was used to enhance embryogenesis, eliminating the need for exogenous phytohormones in the culture media [42].

Palms (Arecaceae Bercht. & J.Presl.) present significant challenges for gene editing. Genetic engineering studies on date palm (*Phoenix dactylifera* L.) are rare and involve complex, time-consuming techniques, such as biolistics and protoplast culture [125,126]. For example, only one gene, *PdSERK1*, has been identified [88] as playing an active role in the embryogenesis of this plant. Indirect studies have also been conducted on the coconut palm (*Cocos nucifera* L.) using genes such as *WUS*, *BBM*, and *SERK* to examine the effects of 5-Azacytidine on explants, as well as its influence on gene expression and somatic embryogenesis [89].

For another economically important monocot, banana (*Musa* L.), direct data on the use of *WUS/BBM* are not yet available. However, research efforts are focused on developing somatic embryogenesis systems where these genes could potentially be applied. While direct introduction of *BBM* or *WUS* into banana has not been reported, progress is being made through optimization of somatic embryogenesis conditions. It is hypothesized that, similar to rice and maize, *BBM/WUS* expression could induce embryogenic callus formation from banana meristems, accelerating the production of transgenic lines [18,44].

Some tropical cereal crops have been influenced by MTFs during in vitro transformation. For instance, in the aforementioned study by Lowe et al. [15], transgenic sorghum and sugarcane plants were successfully obtained using maize-derived *ZmBBM* and *ZmWUS2* in the transformation vectors. The yield of transgenic plants increased from 0–5% to 25–50%. These MTFs promoted somatic embryogenesis in explants. Interestingly, the use of morphogenetic approaches to induce in vitro regeneration is currently more common in monocots, which are generally more challenging to edit compared to dicots. For example, in two cultivars of switchgrass (*Panicum virgatum* L.), another challenging monocot species, a successful case of overexpressing maize-derived *ZmBBM* and *ZmWUS2* has been reported. This approach increased the transformation efficiency of switchgrass to 6% and improved regeneration efficiency to over 40% [90].

For cassava (*Manihot esculenta* Crantz), a crucial tropical crop, researchers have successfully employed grape-derived *VvGRF4-GIF1* and *Arabidopsis AtGRF5*. Their overexpression induced shoot regeneration, increasing efficiency to 50%, though complete elimination of exogenous phytohormones from culture media was not achieved [91]. In cucurbits, including cucumber (*Cucumis sativus* L.), regeneration frequency was enhanced through *AtGRF5* overexpression [92]. Watermelon (*Citrullus lanatus* (Thunb.) Matsum. & Nakai) transformation using the *ClGRF4-GIF1* chimera further demonstrated the broad applicability of this approach [93]. For sugar beet (*Beta vulgaris* L.), *AtGRF5* overexpression in callus cells significantly improved both shoot regeneration and transformation rates [20].

Studies have also been conducted on the application of MTFs in the genetic modification of forest trees. Of particular significance are species that are prized for their timber, their ability to withstand abiotic stress, their resistance to disease, and their potential for reforestation. One such species is eucalyptus (*Eucalyptus* L’Hér.). The treatment of *E. urophylla × E. grandis* ‘DH32-29′ hybrid tissues with *WUS* proteins enhanced the success of transformation and regeneration processes [94].

Several examples of morphogen application have been demonstrated in *Populus* species and cultivars. In *P. tomentosa*, heterologous expression of the *BrBBM* gene from turnip led to the formation of somatic embryos from callus cultures. Calli from leaf explants were constitutively expressed with the i gene, and these calli then spontaneously formed embryoid structures and produced shoots [95,96]. To prevent morphological abnormalities, a gene excision system was employed, where the *BBM* cassette was removed from the genome through heat shock-induced site-specific recombination after embryogenesis initiation.

In a study by Pan et al. [96], the co-expression of *PtWUS* and *PtWOX11* increased callus formation, shoot regeneration, and root formation in poplar. Similar results were achieved earlier through the expression of *WOX11/WOX12a* in poplar [97]. In a study by Pan et al. (96), the co-expression of PtWUS and PtWOX11 increased callus formation, shoot regeneration, and root formation in poplar. Similar results were achieved earlier through the expression of WOX11/WOX12a in poplar (97).

In gymnosperm experiments, the *LEC1* gene family has been studied most extensively. For Norway spruce (*Picea abies* (L.) H. Karst.), the overexpression of *PaHAP3A*, an angiosperm *LEC1* homolog, during zygotic embryo development led to the formation of ectopic somatic embryos. This result is consistent with data from angiosperms, where some cells (e.g., embryonic or meristematic cells) are more prone to embryogenic induction. Additionally, studies have shown that the overexpression of genes that maintain meristems can enhance the natural embryogenic capacity of conifers [98]. However, inducing organogenesis or shoot formation de novo in mature conifer tissue using foreign *WUS/BBM* has proven challenging, with no such studies published [127]. Data on the classical *WUS* and *BBM* applications in conifers is lacking due to the high in vitro complexity of these species.

For other valuable species such as oak (*Quercus* L.) and birch (*Betula* L.), as well as other forest trees, no specific publications on the use of MTFs have been identified. These species are also known to be difficult to regenerate in vitro, and research is ongoing to identify endogenous factors that may enhance their morphogenic potential [128].

MTFs could also be applied to ornamental plants. Many ornamental species are resistant to transformation and in vitro regeneration, including roses, carnations, and orchids [25]. Orchids are particularly attractive targets for improving genome editing protocols. However, current successes are limited to optimizing CRISPR/Cas systems, promoters, and transformation methods (including protoplast techniques) rather than in vitro regeneration and morphogenesis. This techniques applicated to little number of orchid species like *Dendrobium* Sw. [129,130] and *Phalaenopsis* [131]. Applying morphogenic factors could accelerate transgenic plant production in Orchidaceae Juss. Challenging status of orchids in genetic transformation is proved by exist of work with PEG-transformation of different orchids species protoplasts like *Cymbidium* Sw., *Phalaenopsis*, *Paphiopedilum* Pfitzer, *Dendrobium*, *Arundina* (D.Don) Hochr. [132]. No literature exists on morphogen use for aquatic ornamental plants.

No reports exist on MTF application in transgenic medicinal plant production. This approach could be promising, as most medicinal plants exhibit poor in vitro regeneration due to high phenolic compound and secondary metabolite content in their tissues, which hinders regeneration processes [133,134,135].

The potential effectiveness of morphogens in spore-bearing and gymnosperm plants presents a particularly interesting field for speculation. Current literature contains no reports on artificial overexpression or knockout of morphogens in mosses, lycophytes, ferns, cycads, Gnetales or *Ginkgo* L. Only data on key morphogen identification in some representatives of these groups, such as bryophytes [99] and certain ferns [136], are available.

## 4. Engineering Principles of Morphogenetic Factor Expression Systems

Incorporating morphogenetic factors into transformation constructs requires careful attention to their configuration and expression control. Unregulated constitutive expression of these powerful regulators can lead to developmental abnormalities and regenerant mortality [13]. Recent years have seen the development of several strategies for creating safe and effective MTF-containing constructs (Table 3).

### 4.1. Transient Expression (Non-Intergrated)

One approach involves ensuring short-term gene expression without genomic integration by placing the gene cassette outside the T-DNA boundaries [13]. In *Agrobacterium*-mediated transformations, such cassettes typically do not integrate (due to the termination of the T-DNA boundary) but remain temporarily active. For example, constructs containing *WUS2* and *BBM* positioned outside the left T-DNA border in maize allowed the formation of embryogenic structures without stable transgene integration, enabling the transformation of previously resistant genotypes [15].

### 4.2. Chemically Inducible Promoters

Chemical induction systems (e.g., dexamethasone-, tetracycline-, or estrogen-responsive promoters) allow precise temporal control. The MTF remains inactive until induced, minimizing pleiotropic effects. For instance, *Arabidopsis* constructs with *35S::*BBM*-GR* (glucocorticoid-receptor fusion) enabled dexamethasone-inducible embryoid formation on hypocotyls [39,40]. While underutilized in routine transformation due to protocol complexity and variable inducer penetration, these systems show experimental efficacy [13].

### 4.3. Tissue-Specific Promoters

Spatial restriction using promoters active only in target tissues (e.g., callus or embryoids) reduces off-target effects. Corteva’s maize system employed the *PLTP* (plastid lipid transfer protein) promoter—active during early embryogenesis but silent in seedlings—to drive *WUS/BBM* expression, yielding normal regenerants [15]. Such precision enhances viable transgenic plant recovery.

### 4.4. Gene Excision Systems

Several primary strategies have been established and should be explicitly discussed:

1. Site-specific recombinases: Heat- or stress-inducible *Cre/loxP* systems in maize are commonly used in MTF pipelines to excise *Wus2/BBM* after embryogenesis. This approach mitigates pleiotropy and preserves efficiency [15]. Similarly, a *FLP/FRT* approach was used to remove *BBM* modules in *Populus* tomentosa after a brief heat shock, restoring normal growth [119]. In hexaploid wheat, the *moCRE/loxP* system excised both morphogenic and marker cassettes in the “QuickWheat” system, yielding high-quality events without MTFs in the final product [137]. Double-T-DNA and segregation strategies: Delivering MTFs on a separate T-DNA molecule allows for simple genetic segregation to remove them from the trait locus in T1 families. This is particularly useful when the goal is gene editing rather than stable MTF expression [15]. For workflows and trade-offs across crops, please see the targeted methodological descriptions in the maize and sorghum MTF papers (see the review in [13] for details). (iii) Non-integrating or transient delivery. Non-integrating *WUS2* vectors can induce embryogenesis without a stable insertion, significantly reducing the burden of cassette cleanup in *Zea mays* [138]. “Altruistic” helper constructs that only express MTFs in a subset of cells can also catalyze regeneration, while being excluded from most recovered events in *Sorghum bicolor* (methodological proof-of-concept; primary report) [31]. (iv) Activation instead of integration. CRISPR-Combo platforms combine editing with transient activation of endogenous regeneration genes (e.g., *WUS* and *WOX11*). This approach shortens culture time and reduces the need for integrating exogenous MTF cassettes [96]. This approach complements excision systems and is compatible with transgene-free editing strategies.

### 4.5. Combining Multiple Morphogenetic Factors

Co-expression of synergistic regulators (e.g., WUS + *BBM*) requires balanced expression—achieved via separate promoters or self-cleaving peptides (e.g., WUS-P2A-BBM). While maize transformation techniques can benefit from this combination [43], *Arabidopsis* exhibits meristem overproliferation, underscoring species-specific optimization needs.

Practical recipes increasingly lean on limited, early exposure to MTFs (hours–days), tissue-preferred promoters, and excision triggers (heat, desiccation) to exit morphogenic states promptly. In wheat, *TaWOX5*—rather than *WUS2/BBM*—overcame genotype dependence with fewer adverse phenotypes [72], and *GRF–GIF* chimeras boosted regeneration across dicots and monocots while minimizing pleiotropy relative to classic embryogenic MTFs [44]. These “lighter-touch” regulators are attractive when the application tolerates smaller gains in efficiency in exchange for simpler cleanup.

## 5. Limitations, Contradictions, and Failure Modes of Morphogenetic-Factor-Assisted Regeneration

MTFs, such as members of the *WUSCHEL/WOX* family and *BABY BOOM (BBM/BBML)*, can dramatically increase transformation and regeneration rates. However, their misexpression has been shown to cause pleiotropic defects. Classic gain-of-function studies have shown that ectopic expression of *BBM* in *Arabidopsis thaliana* and *Brassica* induces hormone-independent somatic embryos and calli, as well as abnormal, neoplastic growth and changes in leaf/flower morphology [39]. Similarly, chemical-induced overexpression of WUS in *A. thaliana* triggers high-frequency somatic embryogenesis in various tissues, with phenotypes that are closely linked to dosage and timing [30]. In crop plants, constitutive expression of *WUS* often disrupts development. For example, overexpression of *AtWUS* in cotton enhances embryogenesis, but also induces ectopic organogenesis and abnormal structures [29]. These findings highlight both the potential benefits and risks of MTFs in crop plants.

The precision of MTF dosage, tissue specificity, and exposure time are therefore crucial. In maize and related monocots, the co-delivery of *ZmWUS2* and *ZmBBM* has increased transformation efficiency and expanded the genotype scope. However, persistent expression produced stunted or sterile plants if MTFs were not transcriptionally limited or excised prior to maturity [15]. In sorghum, *Wus2*-mediated direct somatic embryogenesis has boosted both transformation and CRISPR editing rates, but the study also emphasized the need to restrict MTF expression to the early regeneration stages to avoid harmful carry-over into whole plant development [139]. At the other end of the spectrum, the wound-responsive factor *WOX13* can inhibit shoot organogenesis. In *A. thaliana*, loss-of-function of *WOX13* has increased shoot regeneration, while its misexpression has impeded it. This underscores the importance of considering developmental context and the specific gene family member involved [34]. Together, these primary studies help to explain the contradictory outcomes reported across different species and plant tissues when MTFs are not properly regulated.

The cellular context also plays a significant role in determining the outcome of MTF activity. *WUS (WOX5)* can reprogram root meristem cells to become shoot-like, but this only occurs within specific hormonal environments and developmental stages, highlighting how mis-targeted expression can lead to abnormal outcomes [69]. In practical terms, limiting MTF expression to specific promoters that are active during early development or in response to wounding (such as those used in maize systems) is key to reducing the risk of pleiotropic effects while still maintaining the benefits of regeneration [15]. Guidance for implementing this approach across different taxa can be found in the focused review by Gordon-Kamm et al. [13], which also provides information on promoter and induction strategies.

Beyond visible morphology, culture- and MTF-driven regeneration can induce persistent epigenetic changes. Methylome maps of regenerated *Oryza sativa* showed widespread, stable loss of DNA methylation across generations, which is linked to deregulated gene expression and is consistent with somaclonal variation [140]. Tissue culture steps leave specific epigenomic “footprints” that differ depending on the treatment, warning that even apparently normal regenerants may have latent epigenetic shifts [141]. In clonally propagated *Elaeis guineensis*, the “mantled” somaclonal variant was traced back to hypomethylation of the transposon-rich Karma element in *MANTLED*. These epimutations are responsible for significant field-level defects [142]. These data support the need for explicit, standardized stability testing, including methylation assays, transgene expression trajectories, and multi-generation phenotyping, in pipelines that utilize MTFs.

Finally, “negative results” and contradictions often arise from the interaction between genotype, explant state, and cassette architecture. In wheat (*Triticum aestivum*), MTF-enhanced protocols have produced significant efficiency gains, but dwarfism or reduced fertility has been observed unless the morphogenic cassette is removed or tightly controlled; excision-based “QuickWheat” variants restore normal growth while maintaining the throughput benefits [137]. Even within the same species, the choice of ortholog (e.g., WOX5 vs. WUS2) and promoter sequence can lead to different outcomes, from success to failure. These findings emphasize the importance of reporting not only success stories but also the frequency and range of MTF-related abnormalities and the specific mitigation strategies used (promoter, dosage, induction, excision triggers).

## 6. The Interplay of Exogenous Plant Growth Regulators and Endogenous Morphogenetic Factors During In Vitro Regeneration

The application of MTFs can be combined with phytohormone-mediated regeneration control. In many cases, morphogenesis genes reduce dependence on exogenous phytohormones. For instance, as noted earlier, *BBM* expression in *Arabidopsis* plants led to somatic embryo formation without requiring auxin or cytokinin supplementation in the medium [13]. Similarly, *WUS*, by inducing meristem cell division, can partially substitute cytokinin function [77]. Experimental protocols often demonstrate that when strong MTFs are present, external hormone concentrations can be reduced. For example, maize transformation media containing *WUS/BBM* utilize lower 2,4-D levels than standard protocols to avoid excessive callus formation [15,69].

On the other hand, even when MTFs are incorporated into genetic constructs, phytohormones often remain essential for successful regeneration. Many protocols still include traditional steps: callus induction on 2,4-D-containing medium (where auxin promotes dedifferentiation while morphogenesis gene expression directs callus toward embryogenesis), followed by transfer to cytokinin medium for shoot regeneration. The use of MTFs allows reduced hormone concentrations or shorter induction periods, though complete hormone elimination remains rare except in specific cases [39].

Another approach combines endogenous and exogenous hormonal stimulation. For example, the *Agrobacterium*-derived ipt gene allows transgenic cells to produce cytokinins autonomously [79,80], while *rolC* expression has auxin-like effects [81,82]. These genes allow for lower concentrations of exogenous phytohormones while creating synergistic regeneration stimuli.

However, excessive cytokinin in the presence of *WUS* can cause excessive meristem formation without proper shoot development. Therefore, some protocols use hormone-lean regeneration media to allow morphogenesis genes to dominate the process and avoid competing morphogenetic signals. When using growth factor regulators (GRFs), which enhance general cell proliferation, slightly elevated cytokinin levels can help steer proliferation towards organogenesis, otherwise undifferentiated cell masses can form. This fine-tuning requires culture-specific optimization [13].

## 7. Emerging Applications of Morphogenetic Regulators in Plant Biotechnology

The integration of MTFs in plant genetic engineering is becoming more widespread. The use of morphogens extends the range of species and cultivars that can be transformed, overcoming barriers to transformation in previously difficult crops. This is especially important for woody plants, medicinal species, and high-yielding agricultural cultivars with low in vitro regeneration potential. Novel combinations of regulators (e.g., *PLT* and *GRF-GIF*) can enable transformation of species that are difficult to transform using classical methods. Researchers expect that further developments and discoveries in this area will make the transformation of difficult crops routine and highly effective [15,45,107].

MTFs significantly reduce the time required to produce transgenic plants by accelerating the regeneration process. Protocols now exist that allow for the production of rooted transgenic shoots from certain tomato and soybean varieties in less than 5–6 weeks, bypassing lengthy callus phases [143]. This enables faster experimentation and reduces costs, while also reducing the need for complex micropropagation equipment, as some stages occur in vivo. These advances democratize transformation technology, making it accessible beyond specialized laboratories [13,103].

The search for novel genes that enhance regeneration continues, with ongoing testing of *WIND1, PLT5*, and variants of *WUS/BBM* from various plants [43,45]. This repertoire is likely to expand to include family-specific factors, such as conifer morphogens [98]. Another promising avenue is the use of synthetic regulators, which are engineered transcription factors that combine domains from multiple natural proteins, such as *WUS-BBM* fusions [43].

Innovative DNA/RNA delivery methods are actively being developed. Key advancements include various techniques, such as ribonucleoprotein delivery to minimize explant damage [144]. Biolistic delivery of MTFs-constructs is particularly promising for monocots, as demonstrated by Lowe et al., who achieved 10x higher regeneration in sorghum using biolistically delivered *WUS* (controlled by a weak nos promoter to prevent necrosis) and *BBM* (driven by a strong maize ubiquitin promoter). While effective for crops like soybean [112,113], biolistics can cause developmental abnormalities [145].

Viral vectors carrying morphogens [16], such as *WUS*, can induce meristems systemically, as demonstrated in tobacco [146], wheat [147,148], and tomato [149]. However, this approach requires the use of Cas9-expressing plants and is genotype-dependent, making it labor-intensive.

Future priorities include improving MTFs-constructs to eliminate residual transgenes and phenotypic abnormalities. Emerging strategies include: non-integrated, transient expression; self-excision systems; and precision regulation (light-inducible/tissue-specific promoters). These approaches aim to use morphogens as temporary stimulants with no genomic or epigenetic effects [13].

Because many MTF-enabled pipelines are ultimately used to generate genome-edited crops, it is important to understand that regulatory treatment depends on the final genomic state, rather than on temporary tools used during tissue culture. In many jurisdictions, current global regulations exempt certain genome edits (SDN-1/2, no foreign DNA) from GMO classification, while other countries, such as the EU, still regulate these products as GMOs under existing rules. Reforms are currently being debated (e.g., the EU’s NGT proposal) [150,151]. Broader policy analyses agree that documentation of the absence of foreign DNA and molecular characterization of edited loci are crucial for dossiers [152,153]. Accordingly, when MTFs are used transiently or excised prior to regeneration, the resulting line’s regulatory status will typically track that of a marker-free edited plant, in countries that distinguish between product-based and process-based methods (for more details, see references [150,153]).

In addition to transformation, MTFs can serve as standalone biotechnological tools. For instance, *WUS/ipt* co-expression can enhance shoot multiplication for biomass production, and ex vivo applications, such as treating saplings with morphogen-bearing Agrobacteria, can produce branched clones. However, these uses require caution to avoid unintended genomic alterations.

## 8. Conclusions

Thus, the application of MTFs to enhance the in vitro regeneration capacity of agricultural crops represents one of the key trends in plant genetic engineering biotechnology. The use of morphogens is already helping to overcome a major limitation in plant biotechnology—the low transformation and regeneration efficiency of many crops. As morphogen-based approaches advance, the list of easily and routinely transformable and regenerable crop varieties is expected to expand encompassing genotypes of soybean, rice, rapeseed, maize, cucurbits, conifers, and other plant species that are currently recalcitrant to in vitro manipulation [13,59,91,111]. Despite successes, many unresolved issues remain. It is crucial to thoroughly study the pleiotropic effects of morphogens before their widespread use. This will help identify the most effective morphogens for specific crops and methods to control their function in plants. Most studies on new morphogens focus more on their application in dicots than in monocots. It may be worthwhile for new studies to include both dicot and monocot crops (e.g., soybean and rice, tomato and maize) as research objects. This would enable comparison of morphogen effects across different crops and accelerate the selection of suitable morphogens for each crop and variety. Overcoming the barrier of genetic transformation in most woody and medicinal plants remains a significant area for research. It is possible that, along with MTFs, gene cassettes could include genes (transcription factors like *MYB, bHLH, WD40* [154]) that block the synthesis of phenols, which hinder regeneration processes. The question of effective delivery methods for MTFs alongside other genes into plants is also relevant. Viral vectors and CRISPR/Cas systems could theoretically facilitate the delivery of large constructs or individual genes. Also, techniques of morphogen’s direct delivery are highly desired in plant biotechnology, which could help to eliminate in vitro routine fully or partly [155]. In the future, combining synthetic biology advances, novel vector systems, and MTFs could lead to a radical enhancement of the genetic potential of agricultural plants [16].

## Figures and Tables

**Table 1:** Functional diversity of major morphogenetic transcription factor groups and their applications in in vitro regeneration of plants.

Group Name of Morphogenetic Factors	Genes	Functions in Plants	Observed Impact In Vitro	References
*WUS (WUSCHEL)*	*WUS*	Maintenance of cell populations in shoot and floral meristems; regulation of meristem differentiation	Overexpression enhances shoot regeneration and meristem growth	[25,26,27,28,29,30,31,64,65,66,67,68,69,70]
*WOX* (*WUSCHEL-related homebox*)	*WOX1*, *WOX2*, *WOX3*, *WOX4*, *WOX5*, *WOX11*	Regulation of cell differentiation (*WOX5* maintains root meristems; *WOX11* controls lateral root branching)	Stimulation organo- and embryogenesis; *WOX11* improves in vitro rhizogenesis efficiency	[32,33,34,35,36,37,38,71,72]
*BBM* (*BABY BOOM*)	*BBM*	Control of embryogenesis; regulation of cell proliferation and embryo development	Somatic embryogenesis induction without exogenous hormones	[39,40,41,42,68,73]
*GRF-GIF* (*GROWTH-REGULATING FACTOR–GIF*)	*GRF1*–*GRF9*, *GIF1*–*GIF3*	Management of shoot and leaf growth; cell proliferation	Improvement of regeneration efficiency by promoting prolific shoot formation	[20,44,45,46]
*PLT* (*PLETHORA*)	*PLT1*, *PLT2*, *PLT3*, *PLT5*	Maintenance of root meristems; regulation of embryo development; control of auxin signaling	Root regeneration; overexpression enhances somatic embryogenesis	[43,74]
*LEC* (*LEAFY COTYLEDON*)	*LEC1*, *LEC2*, *FUS3*	Control of embryo maturation and seed germination; regulation of nutrient storage	Somatic embryogenesis induction and embryoid yield increasing	[15,47,48,49]
*SERK* (*SOMATIC EMBRYOGENESIS RECEPTOR KINASE*)	*SERK1*, *SERK2*	Enhancement of somatic embryogenesis; co-factor in signaling pathways	Enhancement of embryogenic competence; and regeneration efficiency	[25,75,76]
*ESR* (*Embryo Surrounding Region*)	*ESR1*	Processes of endosperm formation and seed coat development	Shoot formation efficiency increasing	[50,51,52]
*WIND* (*WOUND INDUCED DEDIFFERENTIATION*)	*WIND1*	Wound-induced promotion dedifferentiation; regulation of callus formation	Cell division induction and proliferation without phytohormones, improving transformation efficiency	[53,54,55,76,77]
*RKD* (*RWP-RK DOMAIN-CONTAINING*)	*RKD1*, *RKD2*, *RKD4*	Regulation of egg cell differentiation and maintenance of embryogenic totipotency	Somatic embryogenesis induction and callusogenesis	[56,57,58,59]
*DOF* (*DNA-binding One Zinc Finger*)	*DOF3.4*, *DOF5.6*	Development of shoot and root apical meristems	Callus induction	[60]
*ipt* (*isopentenyltransferase*)	*ipt*	Oncogene	Enhancement of shoot formation efficiency	[55,61,62,78,79,80]
*rol* (*root loci*)	*rolB*, *rolC*	Alteration of plant cell hormonal balance	Efficient root or shoot initiation	[13,81,82]
REF1 (REGENERATION FACTOR1)	-	Initiation of callusogenesis via *WIND1* activation in response to wounding	Regeneration efficiency improving via callusogenesis	[63]

**Table 2:** Applications of morphogenetic transcription factors in regeneration of different crops and model plants organisms.

Crop	Cultivar	Genes	Observed Impact In Vitro	Reference
*O. sativa*	ssp. *indica* IRV95	*OsBBM* + *ZmWUS2*	43% regeneration efficiency	[15]
		*ZmBBM* + *ZmWUS2*	27% regeneration efficiency	[15]
	Kitaake	*GRF4-GIF1*	Enhancement of regeneration efficiency 2-fold (from 20 to 42.8%)	[44]
	-	*WOX11*	Enabling root system modifications without affecting shoot traits	[72]
	Cempo Ireng	*OsRKD3*	Enhancement of transformation 23.5-fold, enhancement of somatic embryogenesis by activating a gene network (*AP2/ERF*, *MYB*, *COL*)	[59]
*B. napus*	Topas DH 4079	*BnBBM*	Development of somatic embryos in the absence of exogenous phytohormones	[39]
	BNS3	*AtGRF5*	Increasing shoot regeneration to 19.6% (vs. 3% in controls) without developmental defects	[20]
*G. max*	DN50, DN252, DN254, SN4, SN14, ZJ602	*GmGRF3-GIF1*	Enhancement of regeneration up to 5.5–13.8% (vs. 2–5% in controls)	[45]
	CD215, Jack	*AtGRF5*	Enhancement of shoot regeneration up to 50%	[20]
	Dongnong-50	REF1	Enhancement of transformation and regeneration efficiency 5- and 9-fold respectively	[63]
*S. lycopersicum*	Big Beef	*PLT5*	Enhancement of transformation efficiency up to 13.3%	[43]
		*WUS*	Enhancement of transformation efficiency up to 3.3%	[43]
	Alisa Craig, Castlemart	REF1	Enhancement of regeneration efficiency 3-fold	[63]
*Citrus × sinensis* (L.) Osbeck	Valencia	*L1L (LEC1)*	Somatic embryogenesis induction in one month after *Agrobacterium*-mediated transformation	[83]
	‘Pineapple’, ‘Hamlin’, ‘Sucarri’, ‘Valencia’	*ZmKN1*	Enhancement of transformation efficiency by 3–15 times	[84]
*Citrus reticulata* Blanco	Red, Bendizao	*L1L (LEC1)*	Somatic embryogenesis induction in one month after *Agrobacterium*-mediated transformation	[83]
*Citrus limon* (L.) Osbeck	Eureka	*ZmKN1*	Enhancement of transformation efficiency by 3–15 times	[84]
*Citrus medica* L.	Carrizo	*GRF-GIF*	Enhancement of regeneration efficiency 4.7-fold	[44]
*Malus domestica* L.	Royal Gala	*MdBBM1*	Increasing the frequency of transgenic shoot regeneration to ~31%	[85]
*Vitis vinifera* L.	Cabernet Sauvignon	*VvBBM*	Overexpression enhances somatic embryogenesis to 42.85%	[86]
	-	*GRF-GIF*	Shoot formation efficiency increasing by more than 4-fold	[44]
*Fragaria vesca* L.	Hawaii 4	*VvGRF4-GRF1*	Enhancement of transformation efficiency by more than 2-fold, reaching 40%	[87]
*Theobroma cacao* L.	6–1, ICS1	*TcBBM*	Enhancement of somatic embryogenesis induction 5.5-fold without exogenous hormones	[42]
*Phoenix dactylifera* L.	Deglet Nour	*PdSERK1*	Studying SERK1 expression which is highly expressed during embryogenic competence acquisition and globular embryo formation in culture	[88]
*Cocos nucifera* L.	MI-192-17	*WUS, BBM, SERK, LEC*	Investigation of MTF expression during explant treatment with 5-Azacytidine	[89]
*Sorghum bicolor* (L.) Moench	TX430	*ZmBBM + ZmWUS2*	Somatic embryogenesis induction and enhancement of transformation efficiency from 2 to 18%	[15]
*Saccharum officinarum* L.	CP01-1372	*ZmBBM + ZmWUS2*	Somatic embryogenesis induction and enhancement of transformation efficiency from 2 to 273%	[15]
*Panicum virgatum* L.	Summer, Blackwell	*ZmBBM + ZmWUS2*	Enhancement of transformation efficiency to 6% and regeneration efficiency to over 40%	[90]
*Manihot esculenta* Crantz	60444, NASE	*VvGRF4-GRF1, AtGRF5*	Enhancement of transformation and regeneration efficiency to 50% without exogenous hormones	[91]
*Cucumis sativus* L.	Cu2	*AtGRF5*	Improvement of regeneration efficiency by promoting prolific shoot formation	[92]
*Citrullus lanatus* (Thunb.) Matsum. & Nakai	TC	*ClGRF4-GIF1*	Improvement of regeneration efficiency by promoting prolific shoot formation by 47.02%	[93]
*Beta vulgaris* L.	9BS0448, 1RV6183, 7RV5706H, 8RV6921	*AtGRF5*	Enhancement of transformation efficiency 6-fold and regeneration efficiency to 20.7%	[20]
*E. urophylla × E. grandis*	‘DH32-29′	*WUS*	Enhancement of transformation efficiency 3-fold, embryogenic callus and somatic embryogenesis induction 40%	[94]
*Populus* L.	-	*BBM* *WUS*	Embryogenic callus and somatic embryogenesis induction 39%	[95]
	-	*PtWUS + PtWOX11*	Enhancement of callus induction, shoot regeneration and rhizogenesis efficiency, increase in leaf area to 25%	[96]
	84K	*WUS*	Enhancement of rhizogenesis efficiency 1.5–2-fold	[97]
*Picea abies* (L.) H. Karst	88.22, 61.21	*PaHAP3A*	Somatic embryogenesis induction in maturated embryo 5.5%	[98]
*Physcomitrium patens* (Hedw.) Bruch & Schimp.	-	*LSH1*	Studies on the maintenance of meristematic cell activity	[99]

**Table 3:** Strategies for controlled expression of morphogens in plants.

**Type of Strategy**	**Principle of Function**	**Reference**
Transient Expression (Non-Integrated)	Short-term expression without genomic integration, non-integrated cassettes due to T-DNA border termination	[15]
Chemically Inducible Promoters	The morphogen is expressed under a chemically inducible promoter only in the presence of a specific substance in the medium (e.g., dexamethasone-, tetracycline-, or estrogen-responsive promoters)	[13,39,40]
Tissue-Specific Promoters	The morphogen is expressed under a special promoter only in target cells and tissues (calli or embryoids)	[15]
Gene Excision Systems	Post-regeneration removal of morphogenes via recombinase-based systems (*Cre/LoxP*, *FLP/FRT*)	[76,77,78]
Combining Multiple Morphogenetic Factors	Co-expression of synergistic regulators via separate promoters or self-cleaving peptides for balanced expression	[15,16,43,87,90,91,95,96]
